# Ex Vivo Perfusion Using a Mathematical Modeled, Controlled Gas Exchange Self-Contained Bioreactor Can Maintain a Mouse Kidney for Seven Days

**DOI:** 10.3390/cells11111822

**Published:** 2022-06-02

**Authors:** Natalie Won, Jorge Castillo-Prado, Xinzhu Tan, John Ford, David Heath, Laura Ioana Mazilescu, Markus Selzner, Ian M. Rogers

**Affiliations:** 1 Lunenfeld-Tanenbaum Research Institute, Mount Sinai Hospital, Toronto, ON M5G 1X5, Canada; natalie_won@rogers.com (N.W.); jcastillo@lunenfeld.ca (J.C.-P.); xinzhutan@163.com (X.T.); 2 School of Biomedical Engineering, McMaster University, Hamilton, ON L8S 4K1, Canada; 3 Department of Physiology, University of Toronto, Toronto, ON M5S 1A8, Canada; 4 Department of Chemistry, University of Toronto, Toronto, ON M5S 3H6, Canada; ja.ford@utoronto.ca (J.F.); dave.heath@utoronto.ca (D.H.); 5 Department of General, Visceral and Transplantation Surgery, University Hospital Essen, University Duisburg-Essen, 45147 Essen, Germany; laura.mazilescu@uk-essen.de; 6 Soham & Shaila Ajmera Family Transplant Centre, University Health Network, Toronto, ON M5G 2C4, Canada; markus.selzner@uhn.ca; 7 Toronto General Hospital Research Institute, Toronto, ON M5G 2C4, Canada; 8 Department of Obstetrics and Gynaecology, University of Toronto, Toronto, ON M5G 1E2, Canada

**Keywords:** ex vivo organ perfusion, regenerative medicine, kidney disease, bioreactor, mathematical model

## Abstract

Regenerative medicine requires better pre-clinical tools in order to increase the efficiency of novel therapies transitioning to the clinic. Current monolayer cell culture methods are suboptimal for effectively testing new therapies and live mouse models are expensive, time consuming and require invasive procedures. Fetal organ culture, organoids, microfluidics and culture of thick sections of adult organs all aim to fill the knowledge gap between monolayer culture and live mouse studies. Here we report on an ex vivo organ perfusion system that can support whole adult mouse organs. Ex vivo perfusion of healthy and diseased mouse organs allows for real-time analysis that provides immediate feedback and accurate data collection throughout the experiment. Having a suitable normothermic ex vivo perfusion system for mouse organs provides a tool that will help contribute to our understanding of kidney physiology and disease and can take advantage of the many mouse models of human disease that already exist. Furthermore, an ex vivo kidney perfusion system can be used for testing novel cell therapies, drug screening, drug validation and for the detection of nephrotoxic substances. Critical to the success of mouse ex vivo organ perfusion is having a suitable bioreactor to maintain the organ. Here we have focused on the mouse kidney and mathematically modeled, built and validated a bioreactor that can maintain a kidney for 7 days. The long duration of the ex vivo perfusion will help to advance studies on kidney disease and can rapidly test for new regenerative medicine therapies compared to whole animal studies.

## 1. Introduction

Chronic kidney disease is a global health issue with limited options for treatment. Treatment of kidney disease requires an in-depth understanding of disease initiation, progression and impact on other organs. Increased knowledge will lead to improvements in disease modeling, improved targets for drug screens and improved therapies. Mice have been the mainstay of disease investigation and the primary method for gathering data prior to large animal studies and human clinical trials. But small animal models tend to be ‘black box’ experiments, where real-time visualization and assessment is difficult without invasive procedures that can adversely affect the study. Alternatives to animal models are required. The goal is to provide more complexity to standard cell culture but in a more controlled environment that can be assessed in real time with little disruption to the experiment. Cell culture with primary renal cells derived from patients is helpful but the process used to isolate primary cells results in cell damage, cell loss and induced changes such as de-differentiation. Organ on a chip and 3D bioprinting provide a 3D organ-like entity, but they do not contain all of the cells normally found in the organ and have limited structural similarity [1]. Kidney organoids, containing most of the cells found in a native kidney, can be produced and although specific organ substructures are clearly present, full organization is difficult to achieve. For example, both tubules and glomeruli are found but they are not always contiguous. Furthermore, organoids represent a foetal stage of development, making them suitable for interrogating development and foetal stage disease, but their efficacy is limited for adult disease studies [2].

Ex vivo whole-organ perfusion culture has been explored since the 1930’s, with the majority of the studies using porcine organs because it is easier to cannulate the vessels than mouse or rat organs. Currently, porcine organ studies are mainly focused on modeling improvements to donor-organ maintenance during transportation and storage prior to transplantation. The first porcine-organ studies used hypothermic conditions but with improvements to oxygenation and media, normothermic ex vivo organ perfusion was achieved. Ex Vivo Organ Perfusion (EVOP) systems have been developed for lung, liver and kidney [3,4,5]. The power of this system is revealed by studies that have demonstrated that donor human organs can be maintained on the EVOP system and then be successfully transplanted into patients. The clinical usefulness of EVOP is obvious, but it has also been used successfully as an alternative to large animal studies, where cell therapy, drug testing and disease analysis has been carried out [5]. Organs perfused on the EVOP system can be monitored and assayed in real time providing an opportunity to gain data not available during live animal studies.

Success of the EVOP exposes a need for a perfusion system for mouse studies. Mouse organs require less medium and less space, and the organs are more readily available making the mouse system a practical research tool. Therefore, a bioreactor that meets all of the metabolic requirements of the organ but is simple and easy to use is required. Current available bioreactors are designed for the benchtop, thus requiring oxygenation, a media warmer, water-jacketed tubing, circulating water bath, bubble traps and multiple pumps. The benchtop design is good for experimental observations but limits them to short-term experiments as sterility is an issue and the units are cumbersome to operate and expensive [6]. Successful bioreactors that aid in the study of rodent ovaries, heart, lungs, liver and kidneys have been developed [7,8,9,10] but use rat organs. There are few studies using mouse organs, although mouse is the primary small-animal model with 100′s of established models of human kidney disease available [11].

Our goal was to design, build and validate a simple, easy to use bioreactor that could fit into a standard tissue-culture incubator using atmospheric air and one pump. A successful design that allows for sufficient diffusion of oxygen from the atmosphere into the reservoir would either eliminate the aerator or minimize it sufficiently to incorporate it into the bioreactor as one unit allowing for a compact design.

Mathematical modeling is advantageous as it allows for in silico experimentation before expensive prototypes are built and validated. Using mathematical modeling, we were able to develop a design that optimized the surface area of the medium so that sufficient oxygenation occurred within the bioreactor reservoir using just atmospheric air. This was critical as it eliminated the need for an oxygenator and an oxygen tank making the bioreactor much simpler to use, amenable for scale-up for high throughput use and small enough it can fit into a standard tissue-culture incubator. The mathematical model was validated using mouse kidneys to measure organ oxygen consumption, urine analysis and renal histology. The successful preservation of native mouse kidneys using our perfusion-based bioreactor for 7 days is reported in this study.

## 2. Materials and Methods

### 2.1. Ethics Statement

All experiments were approved by the Animal Care Committee at the Toronto Center for Phenogenomics, Mt. Sinai Hospital. All experiments were performed at the Lunenfeld Tanenbaum Research Institute at Mount Sinai Hospital in Toronto, Canada. All animal work was performed in compliance with the guidelines approved by the Animal Care Committee at the Toronto Centre for Phenogenomics, Toronto, Canada and accordance with ARRIVE guidelines (https://arriveguidelines.org (accessed on 3 September 2020).

### 2.2. Kidney Recovery

Kidneys were recovered from CO_2_ euthanized CD1 adult mice. The renal artery and ureter were cannulated with a blunt 30-gauge needle and thin-walled tubing. The cannulas were then sutured in place, and 2–3 mL of phosphate-buffered saline (PBS) (Sinai Health System, Toronto, ON, Canada) was perfused through the renal artery to remove residual blood.

### 2.3. Bioreactor

The bioreactor design presented in the results section was translated to Auto Cad (Autodesk, San Rafael, CA, USA), and built to specifications at the University of Toronto, Department of Chemistry, Glass Blowing and Metals shop.

### 2.4. System Sterility

The glassware and steel components of the custom bioreactor were first washed with a 1:10 biocompatible detergent solution and then sent for autoclaving. Each component was wrapped in autoclavable paper and placed in 4 L glass beakers sealed with aluminium foil. This prevented damage during autoclaving and ensured components remained sterile. Tubing used in the perfusion system was either autoclaved and then replaced following the experiment or in some cases it was washed with bio-compatible detergent, rinsed, autoclaved and reused. The circuit was one meter of tubing. Tygon (Masterflex, Cat#06460-48, (Sigma-Aldrich, Mississauga, ON, Canada) or Silicone (Masterflex, Cat #ZN-06411-63) tubing was used. The bioreactor is assembled as described in the results section.

### 2.5. Kidney Integration into the Bioreactor

Kidneys were maintained in the bioreactor for 0–9 days (*n* = 27). Day 0 (control) *n* = 3, Day 1 *n* = 1, Day 2 *n* = 5, Day 3 *n* = 4, Day 4 *n* = 5, Day 7 *n* = 6, Day 9 *n* = 3.

The bioreactor and pump were kept in the incubator at 37 °C, 18.6% O_2_ and 5% CO_2_, 100% humidity. The glass chamber vented to the incubator and equilibrated with the atmosphere. The perfusion circuit did not include an oxygenator; the only oxygen source came from the atmospheric oxygen dissolving into the medium collecting in the reservoir. Due to the short span of tubing used (1 m) the oxygen diffusion through the tubing was inconsequential, having no effect on the oxygen levels in the reservoir. After initial experimentation, only silicone tubing was used. Silicone tubing was used to connect the bioreactor ports to the pump. The media reservoir contained 50 mL of media. 5 mL of medium was added to fill the tubing. A Fisherbrand Mini-pump-variable flow (#3-876-1, Thermo-Fisher Scientific, Mississauga, ON, Canada) was used. This pump is sealed and compatible for use in an incubator at high humidity and 37 °C. Oxygen readings were obtained with a Presens Oxygen Sensor/OXY-1 SMA Single Channel Fiber Optic Oxygen Transmitter/FTC-PSt3-YOP-4 mm Oxygen flow-through cell/Polymer Optical Fiber (Presens, Regensburg, Germany). 10% FBS/DMEM (FBS, #12483-020, Gibco, Thermo-Fisher, Mississauga, ON, Canada); DMEM #319-015-CL, Wisnet, St. Bruno, QC, Canada) or REC medium (ATCC #PCS-400-040, ATCC, Manassas, VA, USA) was used as the perfusate for two different experimental groups. Medium was replaced every 24 h under aseptic conditions. Bioreactor contamination tests were performed every day, 24-h recycled medium was removed and incubated in Luria-Bertani (LB) agar to assess sterility. For the positive control, kidneys were fixed directly after cannulation (day 0).

### 2.6. Urine Collection

During perfusion of the kidney in the bioreactor, urine was withdrawn from the ureter cannula. A 1 mL syringe was connected to the ureter port and 0.15 mL of liquid was sampled for urinalysis (*n* = 3). The circulating medium was collected as a control.

### 2.7. Tissue Processing

Following bioreactor perfusion, kidneys were fixed in 10% neutral buffered formalin (NBF), imbedded in paraffin wax, and sectioned for immunochemistry (IHC). Primary antibodies for IHC were LTL (Vector labs, B1325, Newark, CA, USA), E cadherin (R&D, AF748, Santa Clara, CA, USA), Nephrin (R&D System, AF4269, Toronto, ON, Canada), KSP (Novus Biologicals, NBP1-59248, Littleton, CO, USA), AQP2 (Alomone Labs, AQP-002, Jerusalem, Israel). The primary antibodies were diluted in 0.1%FBS/0.1%Triton/PBS at a 1:100 ratio (except for E cadherin which was diluted at a 1:50 ratio). The secondary antibodies used were: Strepavidin 488 (Invitrogen, S32354, Waltham, MA, USA), Donkey anti-goat IgG Cy5 (Abcam, ab6566, Cambridge, UK), Donkey anti-sheep IgG NL557 conjugated antibody (R&D Systems, NL010), Donkey anti-rabbit IgG594 (Invitrogen, A21207), Donkey anti-rabbit IgG488 (Invitrogen, A21206). They were diluted in the same solution as the primary antibodies at a 1:500 ratio. Cell nuclei were stained with DAPI. Hematoxylin and Eosin (H&E) staining was also done to visualize tissue morphology.

Kidney was also stained with Periodic-acid Schiff (PAS) reagent staining. Terminal deoxynucleotidyl transferase (TdT) dUTP Nick-End Labeling (TUNEL) assay was done as previously described [12].

### 2.8. Calculations and Oxygen Level Notes

All calculations are in the results. Note that oxygen partial pressure (pO_2_) or oxygen concentration ([O_2_]) are required for different calculations. Conversions to ppm, which is oxygen concentration is done at the end.

## 3. Results

### 3.1. Calculating the Required Dimensions for the Bioreactor Growth Chamber That Would Provide Sufficient Oxygen for Optimal Kidney Growth and Maintenance

Being able to fit the bioreactor unit into a tissue-culture incubator would eliminate the need for a media warmer, water-jacketed tubing and a second pump. Therefore, the diameter and depth measurement of the reservoir had practical limitations. The diameter and length of the reservoir collection tube used to draw the medium up and into circulation had to be considered so it would not disturb the air:liquid interface and generate air bubbles that could damage the kidney. The depth of the medium, the amount of exposed surface area and the time the medium is exposed to atmosphere air were important factors to consider. We empirically calculated the amount of medium required based on the minimal medium required to supply sufficient nutrients to the kidney over a 24-h period. This time period was chosen as it is optimal to change the media every 24 h. A mouse kidney contains approximately 100 million cells [13]. Empirical data were achieved by using tissue-culture studies with adult primary renal epithelial cells (REC) grown in Corning cell stacks. One stack with a surface area of 636 cm^2^ supported 10^9^ cells and used 500 mL of medium for 24 h. We assumed a linear relationship between the cell number at confluence and volume of medium. Therefore, 100 million cells should be maintained with 50 mL medium/24 h. Based on this volume, we chose a bioreactor radius of 5 cm. This allows for a bioreactor that is small enough to easily handle and move in/out of an incubator and also provides for a large medium surface area to volume ratio.

To calculate the [O_2_] at bottom of the reservoir we must consider that an oxygen gradient forms as oxygen diffuses to the base of the reservoir. Previous studies have assessed these internal gradients within cell culture media and often use Fick’s first law (Equation (1)) or derivations of Fick’s first law to describe oxygen consumption.

Fick’s First Law: F = D X ΔC/ΔD(1)

Fick’s first law describes the flux of oxygen and is based upon the presence of an oxygen source and an oxygen sink. In the referenced study, the [O_2_] in the gas acted as the oxygen source while cellular consumption of oxygen acted as the sink. These two variables were the driving force for the oxygen gradient. Due to the nature of our experimental setup, cellular consumption of oxygen was not considered a sink for the oxygen gradient in the reservoir as the organ or cells are not present in the reservoir. In addition, vascular perfusion of the whole kidney could not be equated to a monolayer of a single cell type. It was assumed that the only significant oxygen gradient would be in the acellular media reservoir and that a constant concentration of oxygen would be delivered to the kidney through the tubing. Following cellular consumption, the media would then have oxygen reinfused in the media reservoir. The recycled media in the reservoir created a transient system, and therefore the simplified version of Fick’s first law could not be applied to model the gradient.

Instead, a complimentary error function was derived to approximate oxygen flux (Equation (2)). Further information on this derivation can be reviewed elsewhere [14]. We want to solve for *y_kidney_*, the concentration of oxygen in the kidney.

Oxygen Concentration Approximation:

(2) $$\frac{y_{kidney}-y_{o}}{y_{liquid}-y_{o}}=erfc\left(\frac{x}{2\times \sqrt{D\times t}}\right)$$

y_liquid_ is the molar concentration of oxygen at the liquid-gas interface. This is calculated from Henry’s law (Equation (3)), which states that the partial pressure of oxygen in the gas phase is proportional to the dissolved oxygen in the liquid medium (*y_liquid_*) immediately below the interface.

Henry’s Law:

(3)  yliquid=pgasH; gas partial pressure (p gas) over Henry′s Constant

Henry’s constant for oxygen diffusion into medium at standard incubator conditions (37 °C and 18.6% Oxygen) was previously determined [15]. Since Henry’s constant (*H*) is unaffected by air pressure differences less than 506 kPa [14,15] and the atmospheric pressure between our conditions and the study’s conditions are similar, a Henry’s constant of H = 771.65 mmHg/mM was used. To obtain $p_{gas}$ the following were used: Incubator conditions =18.6% oxygen, the average atmospheric pressure in Toronto, Canada = 753.66 mmHg, therefore $p_{gas}$ (partial pressure of oxygen (pO_2_)) in the incubator air = 753.66 mmHg × 0.186= 140.18 mmHg. Therefore, using Henry’s law, the dissolved gas (y_liquid_) just below the liquid interface = 140.18 mmHg/771.65 mmHg/mM = 0.181 mM = 5.8 ppm.

The height of the 50 mL volume was calculated from: Height of a Cylinder: $h=\frac{V}{\pi \times r^{2}}$ .

This results in a total height of the medium of 0.667 cm. The collection tube is 0.267 cm from the bottom, therefore it is collecting medium from a depth of 0.40 cm.

*x* (medium depth) = 0.40 cm.

D (Diffusion Coefficient) =2.84 × 10^−5^ cm^2^/s.

H (Henry’s Constant) = Toronto Atmospheric Pressure: P = 100.48 kPa = 753.66 mmHg.

Incubator Oxygen Content (18.6%): pO_2_ = 132 mmHg @ 37 °C.

yliquid=pgasH ; $y_{liquid}$ = 132 mmHg/771.65 mmHg/mM = 122.4 mmHg oxygen.

The oxygenation at depth was calculated for two medium renewal rates. Medium renewal is the time it takes for all of medium in the reservoir to flow through the system once. ∆t = 2730 s is close to physiological renal flow rates for mouse and ∆t = 9091 s was calculated to determine if giving the medium more time in the reservoir increased oxygenation.

(a) Δt = 2730 s. and (b) ∆t = 9090 s.

Oxygen Concentration Approximation

$\frac{y_{kidney}-y_{o}}{y_{liquid}-y_{o}}=erfc(\frac{x}{2\times \sqrt{D\times \Delta t}}$ )

(a)
$\frac{y_{kidney}-0}{0.171\mathrm{mM}\;-\;0}=erfc(\frac{0.4}{2\times \sqrt{2.84\times 10^{-5}\times 2730}}$ )
$\frac{y_{kidney}-0}{0.171\mathrm{mM}\;–\;0}=erfc(\frac{0.4}{0.5538}$ )
  Supplementary Table S1; erfc (0.7222) ≈ 0.3086

$y_{kidney}$ = 0.171 × 0.3086 = 0.0528 mM = 40 mmHg = 1.69 ppm

(b)
$\frac{y_{kidney}-0}{0.171\;\mathrm{mM}\;-\;0}=erfc(\frac{0.4}{2\times \sqrt{2.84\times 10^{-5}\times 9090}}$ )
$\frac{y_{kidney}-0}{0.171\mathrm{mM}\;-\;0}=erfc(\frac{0.4}{1.1016}$ )
  Supplementary Table S1; erfc (0.3631) ≈ 0.6107

$y_{kidney}$ = 0.171 × 0.3631 = 0.0621 mM = 47 mmHg = 1.98 ppm

Under physiologic conditions an oxygen gradient is formed between the blood and the tissue. The oxygen content in the blood is maintained at 0.130 mM, and this value then drops to 0.052 mM (40 mmHg = 1.69 ppm) at tissue level [15]. The theoretical oxygen diffusion achieved by the bioreactor reservoir for both medium renewal rates falls within the range of physiological conditions at tissue level.

### 3.2. Bioreactor Assembly

**Figure 1:**
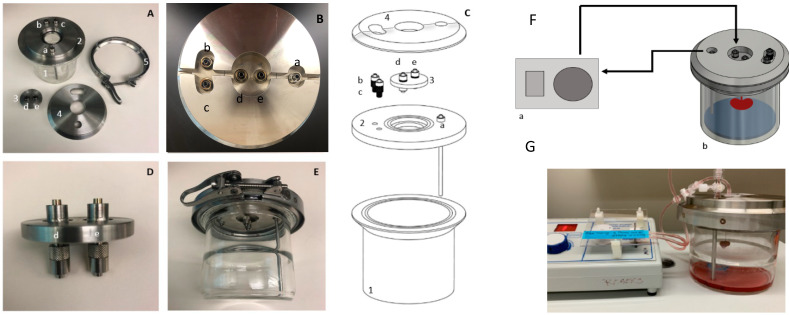
Kidney Bioreactor. Detailed views of the mouse kidney perfusion-bioreactor and individual components (**A**) Unassembled bioreactor with labelled ports: (a) media collection port, (b) adjustable vacuum port, (c) gauge, (b,c) may be replaced with sterile air filters for chamber ventilation, (d,e) custom swivel male Luer ports for renal artery and ureter cannulas. (**B**) Top view of assembled bioreactor. (**C**) Exploded schematic of bioreactor. (**C1**) Glass media chamber. (**C2**) Base lid. (**C3**) Kidney suspension plate. (**C4**) Alignment plate. (**D**) Magnified image of the kidney suspension platform. The suspension platform has two regular male luer locks on the top side and two custom swivel male Luer locks below. (**E**) Interior view of the full assembly. (**F**) Diagram of perfusion circuit setup. (**G**) Perfusion circuit setup with a singular adult mouse kidney.

The bioreactor consists of four primary components—the main chamber, the base lid, the kidney suspension plate, and the alignment cap (Figure 1A–E). The main chamber is a flanged glass jar (Figure 1(C1)) that houses the suspended kidney during long-term cell culture. The chamber has an inner diameter of 10 cm and a height of 10 cm. The lid of the chamber is assembled from three separate steel components. The first component contains the functional ports and is the base for the lid assembly (Figure 1(C2)). The base lid includes a long tube to collect the recycled media from the reservoir, an opening for the kidney suspension plate and additional ports. Integrating a steel media tube prevents tube movement in the bioreactor, which prevents damage to the kidney. Previous iterations of the design involved a silicone collection tube within the chamber [16]; however, the natural twisting of the tubing often damaged the kidney during assembly.

Each opening has embedded metal male luer locks so that the bioreactor is compatible with 3/32” tubing. The luer locks also act as a port site for media analysis and replenishment. The second component of the lid assembly is the circular kidney suspension plate (Figure 1(C3)), a separate plate that only attaches to the kidney and allows for unobstructed addition/removal of the kidney. The suspension plate is 45 mm in diameter and has two sets of connected male luer locks. The top of the plate has two standard male luer locks that are 1.8 cm apart and then these standard locks connect to two custom swivel male luer ports below to create two ducts through the platform. The custom ports attach to the cannulas of the renal artery and vein or ureter. The swivel luer locks prevent twisting of the cannula and reduce damage to the kidney during handling. This suspension plate sits within a central ledge engraved in the base lid. The third component is the alignment piece (Figure 1(C4)). It sits above both the base and suspension plate and aligns the components with the main glass chamber. The alignment piece seals the gap between the suspension plate and the base and provides a platform for clamping. The alignment plate is in two halves, which allows for removal without having to disconnect any of the tubing attached to the kidney. At each junction, an O-ring is inserted to create an air-tight seal. All components are clamped together with a ring clamp (Figure 1E) to maintain an isolated system. The final setup, with the pump, is then placed into the incubator (Figure 1F,G). The assembly of the bioreactor is performed in a biological safety cabinet to maintain sterile conditions.

### 3.3. Validation of the Mathematical Model

To confirm that the bioreactor could support an isolated kidney we attached cannulated mouse kidneys to the bioreactor and measured artery and venous oxygen, urine composition directly from the ureter and histo-pathology using H&E and IF. The higher protein content of the 10% FBS/DMEM medium was more suitable to measuring protein reabsorption than the REC medium, so 10% FBS/DMEM medium was used for all oxygen measurements and urine experiments. Histo-pathology was done on kidneys grown in both media. Media did not contain hemoglobin or oxygen carriers, so the oxygen calculations are applicable to both media. In order to obtain experimental values for oxygenation, reading of oxygen levels in the medium were taken with a Presens oxygen meter. To determine if the bioreactor could re-oxygenate the venous medium sufficiently, the renal artery of the ex vivo adult mouse kidney was cannulated and connected to the bioreactor with the oxygen sensor situated just before the renal artery-cannula connection.

**Figure 2:**
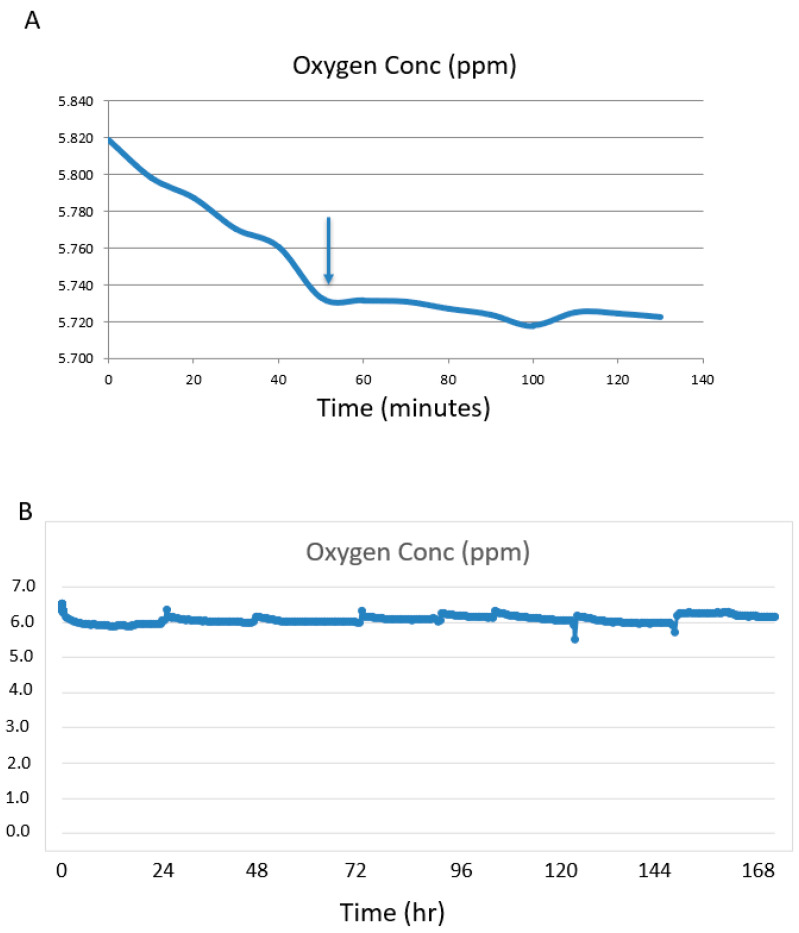
Changes in oxygen concentration of the media at the beginning and end of a 24 h ex vivo perfusion cycle. (**A**) Oxygen levels of the medium before the kidney is attached starts at 5.82 ppm at time =0 at 4 °C and levels off at ~5.7 ppm after 50 min at 37 °C (arrow). (**B**) A mouse kidney was cannulated and perfused in the ex vivo perfusion system for 7 days. The oxygen levels were held to a narrow range of 5.7–6.3 ppm, indicating that the media was well-oxygenated throughout the kidney perfusion period.

The flow rate was set at 1.1 mL/minute. The system was first run without a kidney attached to determine the time for the [O_2_] to stabilize. The medium in the bottle stored at 4 °C was oxygenated as the oxygen level at time = 0 was 5.82 ppm (Figure 2A). During the cycling period through the bioreactor at 37 °C the [O_2_] stabilized a little lower at 5.74 ppm at ~50 min (Figure 2A). This oxygen concentration was higher than the calculated amount of 2.0 ppm.

Silicone tubing is porous to oxygen and can act as an oxygenator. Increasing the length of the tubing between the pump and the bioreactor will increase the oxygen in the medium if saturation has not been reached and will decrease the time to saturation. In a series of non-kidney experiments we tested 1 m and 6 m of silicone tubing and compared this to 6 m of Tygon tubing. Tygon tubing was used as a control as it is not permeable to oxygen. There was no difference in [O_2_] for all parameters tested. The [O_2_] for silicone tubing was the same for 1 m and 6 m lengths and for the Tygon tubing (6 m), indicating oxygen saturation of the medium was achieved through diffusion of medium in the reservoir. For all tubing types and lengths, the medium oxygenation levels were ~5.7 ppm, so 1 m of silicone tubing was used for all experiments.

When the mouse kidney was attached to the system, the medium oxygen concentration held at a narrow range of between 5.7 ppm and 6.3 ppm for the 7 days (Figure 2B). Fluctuations were observed during media changes or if the bioreactor was repositioned, as the sensor is sensitive to motion. These are seen as spikes at 24 h intervals—the times the media was changed.

To measure the oxygen consumption of the kidney, the venous medium was sampled and measured. The venous O_2_ was 4.4 ppm (4.2–4.6 ppm) for the duration of the experiments. Oxygen consumption was calculated and adjusted to ppm/mL/gram of kidney.

(Oxygen_Artery_ − Oxygen_Vein_)/mL/gram of kidney weight:

Oxygen_A_: 5.73 ppm

Oxygen_V_: 4.40 ppm

Flow rate (mL/min): 1.1

weight of kidney: 150 mg

$=\frac{\left(\frac{5.7-4.4}{1.1}\right)}{\left(\frac{1000\mathrm{mg}}{150\mathrm{mg}}\right)}$

=1.18 ppm/mL/0.150 g (per kidney)

=6.77 ppm/mL/gram (adjusted to gram of tissue).

The calculations determined that the bioreactor built to our calculated specifications and run near the physiological flow rate (1.1 mL/min) would provide sufficient medium oxygenation to meet the physiological tissue requirement of 40 mmHg = 1.7 ppm. Interestingly, the actual reading of [O_2_] in the medium near the bottom of the reservoir where the intake tube draws up the reservoir medium, at a flow rate of 1.1 mL/minute, was 5.73 ppm. This was about the same as the calculated amount for the [O_2_] at the medium surface (5.8 ppm). This could be explained by an unaccounted mixing of the medium at the bottom with surface medium as it is drawn up into the collection tube causing increased oxygenation.

Our goal was to achieve physiological [O_2_] levels in the medium. The experimental arterial reading was 5.73 ppm and exceeded the physiological oxygen levels of arterial blood at the tissue level in mice of 40 mmHg = 1.7 ppm. Importantly, our reading at the vein cannula was 4.2–4.6 ppm, which is above the minimum physiological requirement, indicating the kidney was well oxygenated.

### 3.4. Urine Analysis

Because the kidney is cultured ex vivo without the influence from other tissues or organs, the urine production is in the form of ultrafiltrate and does not contain all the components of normal urine. For example, there is no muscle in the system, so creatinine is not produced and therefore does not appear in the ultrafiltrate. We used 10%FBS/DMEM-high glucose as the medium for these experiments. This medium contains high levels of total protein, albumin and glucose, and thus we could test the efficiency of the kidney to reabsorb proteins and glucose better than with REC medium, which contains lower amounts of each of these components.

If the ex vivo perfused kidneys were functioning properly, it was expected that there would be a decrease in the amount of protein and glucose from the fresh medium (t = 0) compared to perfusion medium at 20 h. Fresh 10%FBS/DMEM/high glucose medium contained 386.82 mg/dL of total protein and 3.23 mg/dL of albumin. The reservoir medium after 20 h of circulation through the kidney contained 305.55 mg/dL of total protein and 2.87 mg/dL of albumin, indicating the kidney consumed nutrients as expected.

Furthermore, if the ex vivo kidney was functioning properly the functioning glomeruli would filter out the protein followed by reabsorption of the proteins by the proximal tubules. This would result in a low protein concentration in the urine compared to the medium. It is also expected that the high glucose in the medium would result in the movement of glucose from the perfusion medium to the ultrafiltrate being produced by the glomeruli, but a properly functioning kidney should then reabsorb the glucose so the excreted urine glucose levels would remain low. For urine analysis the ureter was cannulated and 0.15 mL of fluid was collected at day 7 of ex vivo perfusion. The perfusion medium, acting as the ‘blood’ (control 2), is considered input and was collected at the same time urine was collected from the ureter cannula. The medium was changed every 24 h, and the input perfusion medium and ureter ultrafiltrate were collected ~20 h post medium change at day 7 (Table 1). The reservoir medium at the time of urine collection (t = 20 h) had 305.55 mg/dL of total protein and 2.87 mg/dL of albumin while the total protein concentration in the ultrafiltrate collected from the ureter at the same time contained 18.76 mg/dL of total protein (range 9.68–27.84) and 0.06 mg/dL of albumin (range 0.01–0.11), indicating that the majority of the protein was reabsorbed as expected for a functioning kidney (Table 1). Glucose levels were measured at the same time protein was measured. Glucose is freely filtered by the glomeruli but is reabsorbed in the tubules by SGLT1 and SGLT2 [17]. At twenty hours post medium renewal on day 7, the circulating medium contained 399.78 mg/dL of glucose. In the ultrafiltrate the glucose was 4.08 mg/dL, about 1% of the levels in the perfusion medium, indicating the kidney was functioning correctly and reabsorbed the excess glucose accumulating in the ultrafiltrate.

### 3.5. Physical Characterization of the Bioreactor Grown Kidneys

**Figure 3:**
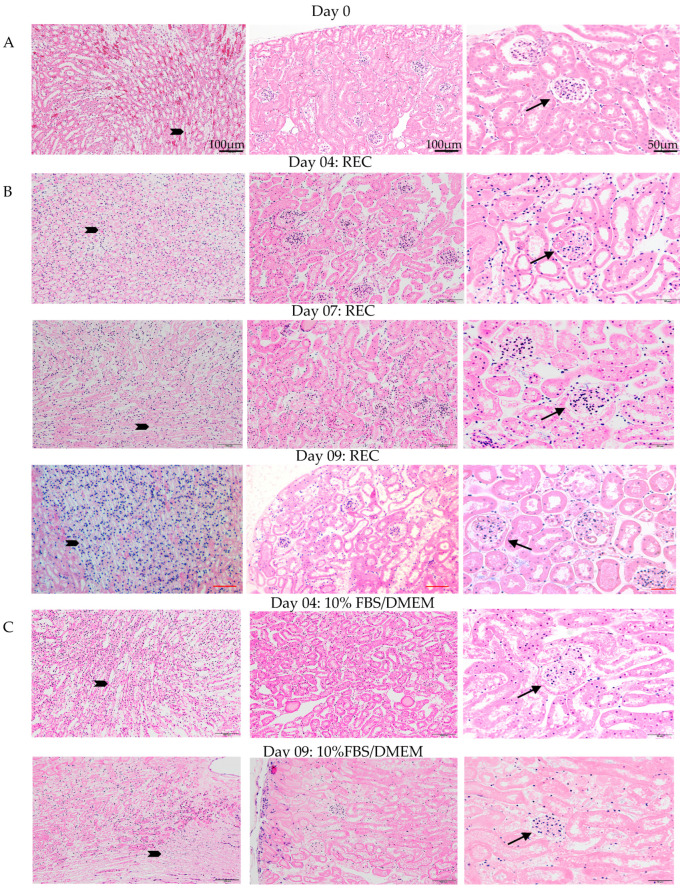
Kidney pathology post ex vivo perfusion. (**A**) Control kidney, cannulated but not perfused. (**B**) Kidneys perfused with Renal Epithelial Medium for 4, 7, 9 days have similar pathology and intact nuclei to control kidneys. (**C**) Kidneys perfused with 10% DMEM medium for 4, 9 days. Day 4 have healthy pathology but some degradation with weak nuclei staining at day 9. Arrows indicate glomeruli and block arrows indicate collecting ducts. Bar = 100 µm for first two columns and 50 µm for last column. Representative bar in top row.

Oxygenation levels of the medium during ex vivo perfusion exceeded physiological levels, and urine analysis indicated that the kidney is healthy and functioning. Histological examination of the kidneys by H&E and immunofluorescence (IF) was carried out to confirm the physical integrity of the kidney post ex vivo perfusion. Ex vivo perfusion used either 10%FBS/DMEM or specialized renal epithelial cell (REC) media. H&E staining was done to assess kidney morphology (Figure 3). Day 0 control kidneys were fixed and processed directly after cannulation. All three time points for kidneys grown in REC medium (day 4, 7, 9) have a similar morphology to that of the day 0 controls. The cells look healthy with healthy nuclei and no signs of oedema or necrosis. The day 4 10%FBS/DMEM grown kidney also resembled the day 0 control kidney. The kidney grown for 9 days in 10%FBS/DMEM had good morphology but was showing signs that tissue degradation was beginning as fewer healthy nuclei were observed in the tubules. Representative magnification bars are in the first panel for each column (Figure 3).

**Figure 4:**
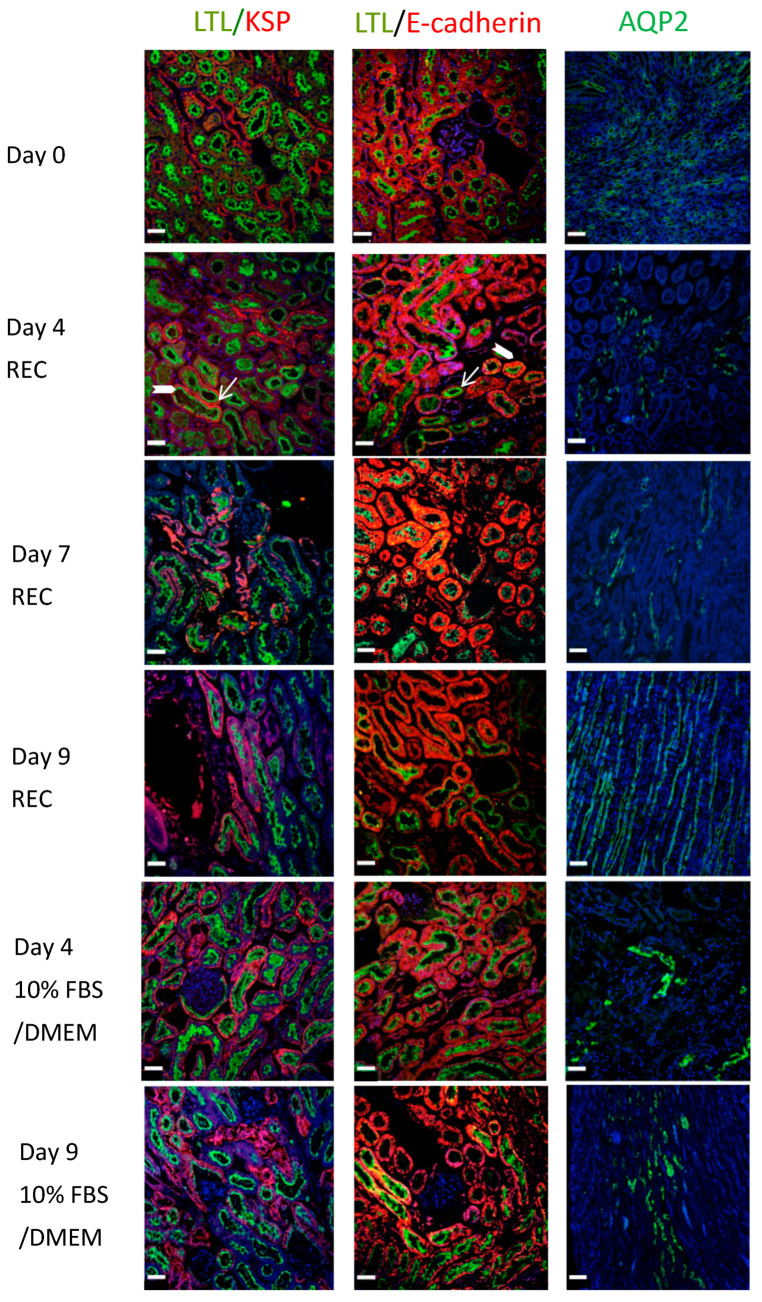
Tubule and collecting duct staining depicts healthy kidneys. Control kidney, cannulated but not perfused. LTL (green), KSP (red) double positive depict proximal tubules. E-cadherin (red), double positive with LTL depicts distal tubules. AQP2 (green) depict collecting ducts. Kidneys perfused with Renal Epithelial Medium for 4, 7, 9 days demonstrated healthy tubules and collecting ducts. Kidneys perfused with 10%FBS/DMEM for 4, 9 days demonstrate healthy tubules and collecting ducts. Day 9 morphology is intact. Block arrows = LTL, Arrow = KSP or E-cadherin. Bar = 45 µm.

Renal sections were also co-stained for LTL (green) and E-cadherin (red). E-cadherin surrounds the tubule cells (Figure 4, block arrow: Day 4 REC: LTL/E-cadherin) while LTL is mainly on the apical side, seen here located as an inner ring in cross-sections of tubules (Figure 4, arrow: Day 4 REC: LTL/E-cadherin). E-cadherin is abundant in the distal renal tubules and its expression is low in the proximal renal tubules. Its co-expression with LTL depicts proximal tubule cells. AQP2 (green) highlights collecting ducts. The ducts are more numerous in the medullary region (Figure 4: Day 9 REC: AQP2) compared to the cortical regions (Figure 4: Day 4 DMEM: AQP2 and Day 7 REC: AQP2). All data points demonstrate strong staining and excellent tissue morphology. Despite the onset of tissue decline observed with H&E staining of the day 09 kidney perfused with 10%FBS/DMEM. The IF staining looked similar to the day 0 controls for all proteins.

**Figure 5:**
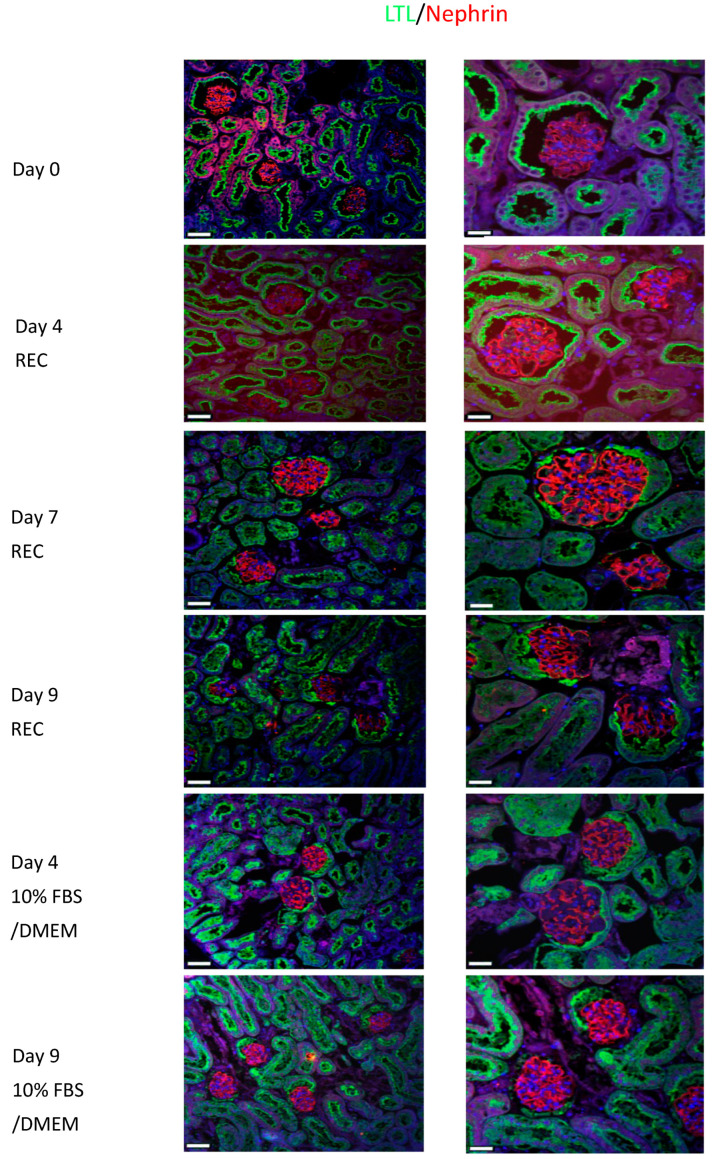
Intact healthy glomeruli observed in all conditions. Control kidney, cannulated but not perfused. LTL (green), Nephrin (red). LTL marks proximal tubular epithelial cells, and the parietal epithelial cells of urinary pole of the Bowman’s capsule. Nephrin depicts podocytes. Kidneys perfused with Renal Epithelial Medium for 4, 7, 9 days demonstrate normal Nephrin patterns indicating healthy glomeruli. Kidneys perfused for 4, 9 days also demonstrate Nephrin expression indicative of healthy glomeruli. Low mag bar = 45 µm, High mag bar = 23 µm.

Nephrin (red) is localized to the podocytes and has a distinct staining pattern that can be clearly observed in the glomerulus of the day 0 control kidneys (Figure 5). All REC and 10%FBS/DMEM cultured kidneys show similar staining to the control kidney. LTL (green) is used as a counter stain. Nephrin is important for maintaining the slit diaphragm and it is important for proper kidney function.

## 4. Discussion

There are no commercially available bioreactors designed for mouse organs for long-term organ culture. We set out to design and test our own system by first mathematically modeling a small system that would easily fit within a standard tissue culture incubator and not require external oxygen or an oxygenator. Parameters that had to be considered during the design stage of the bioreactor were the surface-to-volume ratio of the medium in the reservoir, flow rate of the medium through the kidney and oxygen consumption while keeping the bioreactor small and easy to handle. It was possible to optimize the surface area to volume of the medium to maximize oxygen diffusion, but we also had to account for the oxygen consumption of the kidney. High consumption could lower the oxygen concentration in the medium such that the flow rate to recirculate the medium would have to be slowed down to allow for sufficient time in the reservoir to obtain full oxygenation, but the slow flow rate may not sufficiently support the kidney. A literature search of the oxygen consumption of a kidney in a live animal, kidney oxygen levels and flow rate was used to model the bioreactor. We also took into consideration that exogenous hemoglobin was not being used as we were aiming for a simple bioreactor system. Engler et al. [8] used mathematical modeling to optimize a bioreactor for rat lung. The lung has advantages as it can be its own oxygenator whether air or liquid is pumped through the trachea coupled with oxygen diffusion in the reservoir medium [18]. The liver also has advantages as it obtains oxygenated blood from two inputs, the portal vein and the hepatic artery. The kidney is susceptible to low oxygen and the podocytes in the kidney cortex consume more oxygen than cells in the medulla, so we had to provide for the most oxygen-sensitive areas of the kidney.

In order to meet the metabolic requirements of a whole functioning kidney, a suitable flow rate for oxygenated medium is required. A fast flow rate of oxygen rich medium will be suitable, but counter to this, the venous oxygen-depleted medium requires sufficient time in the reservoir to be replenished by diffusion. Using a rat lung, Engler et al., 2018 [8], demonstrated that media reached an oxygen saturation equilibrium of ~140 mmHg in 20 min when the medium flow rate was 27 mL/min and 60 min with a flow rate of 3 mL/min. This rapid rate of equilibrium was reached due to the lung acting as its own aerator and the use of a PDMS hollow fiber cartridge that provided a surface area equivalent to the alveolar surface area of a rat lung, approximately 2500 cm^2^. The mouse kidney is smaller than a rat lung and requires less oxygen. Without a kidney attached, the medium in our bioreactor equilibrated to an [O_2_] = 5.73 ppm in 50 min. We demonstrated that by increasing the surface area of the medium by passing it through 6 m of silicone tubing there was no significant increase in the time to reach equilibrium or on the final level of oxygenation of the medium, indicating the oxygen diffusion directly over the medium surface area in the reservoir was sufficient.

With a kidney attached and using a flow rate of 1.1 mL/min and 1 m of silicone tubing we observed the O_2_ concentration at the artery cannula of between 5.7 ppm and 6.3 ppm for the 7 days. Medium changes at 24 h would allow for mixing of the air and medium, which would cause a spike in oxygen levels followed by a small decrease to equilibrium levels. Taken together the medium remained at or near saturation levels throughout the seven-day experiment. Medium was collected from the venous cannula and oxygen levels were measured. O_2_ concentration ranged from 4.2–4.6 ppm coming from the venous cannula. It is important to note that the depleted venous medium still had an O_2_ concentration over the minimum physiological requirement (2 ppm) required for maintaining a healthy mouse kidney.

Analysis of the kidney through histology and urine collection from the ureter indicated that kidneys remained healthy for up to 7 days in ex vivo perfusion. Note that the ex vivo kidney produced ultrafiltrate, which is a good indicator of function but does not contain all of the components of urine as the kidney is isolated from muscle and other organs that contribute to urine composition. We could still measure protein and glucose reabsorption, which are good indicators of glomeruli and tubule function. A normal functioning kidney filters proteins and glucose in the glomeruli that are then reabsorbed by the proximal tubules along with glucose. An intact glomeruli basement membrane (GBM) and healthy podocytes act as a barrier to prevent albumin from entering the urine. Other proteins that can pass through the GBM are then reabsorbed by the proximal tubule epithelial cells, resulting in low levels of filtered proteins in the filtrate beyond the proximal tubule [17,19,20]. Furthermore, the metabolism of a healthy kidney would result in the utilization of glucose and proteins, and a decrease of glucose and proteins in the reservoir medium should be observed [17]. The levels of total protein in the urine were 6% of the levels found in the circulating medium, and the albumin in the urine was <1% of the amount in the medium, indicating proper function with the ex vivo perfused kidneys at day 7. Furthermore, high levels of glucose in the medium would be filtered through the glomeruli and then reabsorbed by the tubules resulting in low glucose in the ultrafiltrate [17]. The kidneys can reabsorb glucose, but at 11 mmol/L in the blood the glucose will begin to build up in the urine. At a blood glucose level of 25 mmol/L reabsorption by the tubules is maximized, and as glucose levels rise beyond this level so will the glucose in the urine [17]. Using medium with high glucose we observed very low levels of glucose in the urine (0.20 mmol/L), indicating the kidney remained functional at day 7 of ex vivo perfusion.

Since there is no bladder and no muscle tissue to help control or promote urine flow there was a possibility that urine would not enter the ureter cannula and could not be easily collected. Urine did enter the ureter cannula despite its upward trajectory, indicating the vasculature pressure was large enough to allow for urine to flow into the ureter catheter. It is possible that during the experiment some of the urine backed up into the kidney, which would potentially cause tissue damage. Changing the catheter to the down position should prevent urine backup and should help achieve healthy kidneys beyond day 7. For future experiments, the ureter catheter will be repositioned to the down position. This will require adding a port near the bottom of the bioreactor just above the medium.

Pathology analysis demonstrated that for long-term culture the specialized REC medium was superior to using 10%FBS/DMEM. Using a combination of H&E staining, immunofluorescence for mature renal proteins, Periodic-acid stain, TUNEL and urine analysis, our data indicated that our bioreactor could maintain a functioning kidney for 7 days but organ stress could be observed. The kidneys perfused with the 10%FBS/DMEM demonstrated normal tissue morphology and normal protein expression patterns as indicated by H&E and IF during 7 days of ex vivo perfusion, but by day 9 of perfusion the H&E staining showed areas of tissue degradation, whereas the kidneys perfused in the REC medium showed normal H&E staining. Immunofluorescence to detect LTL, E-cadherin, KSP, Nephrin and Aquaporin 2 demonstrated similar expression patterns between the control kidneys and ex vivo perfused kidneys for both REC medium and 10% FBS/DMEM medium. Intact tubules expressed LTL on the apical membrane of the epithelial cells as expected, indicating healthy kidneys. Double positive E-cadherin and LTL demarked mature and healthy tubules. Aquaporin-2, a marker for the collecting duct, had similar expression in the control kidney and the ex vivo perfusion kidneys. Staining with nephrin, which demarcates the podocytes and produces a distinct pattern in healthy tissue, we observed strong nephrin expression with correct morphology for all ex vivo parameters tested, even in day 9 10%FBS/DMEM kidneys. Periodic-acid Schiff staining demonstrated intact brush borders on renal tubular epithelial cells with similar staining for both day 0 and day 7. Interestingly, TUNEL staining, which detects double-strand DNA breaks and indicates programmed cell death, showed that even though all other indicators suggested a healthy kidney at day 7 of perfusion, the organ had some positive TUNEL staining indicative of organ stress. Considering that urine analysis indicated good kidney function at day 7 of ex vivo perfusion, as we observed the kidney’s ability to reabsorb high levels of protein and glucose, the TUNEL staining in the same kidneys tells us that the kidney was beginning to be stressed. The design of the bioreactor ensures sufficient oxygenation of the mouse kidney, so to improve kidney health during long perfusion periods we will focus on improving the medium with nutritional supplementation.

## 5. Conclusions

Our study demonstrates that ex vivo perfusion of a mouse kidney can be successful for up to 7 days. This model highlights the importance of a dynamic system that mimics the in vivo environment. Constant flow supplied by the perfusion-bioreactor is vital for supplying sufficient nutrients and oxygen to the tissues as well as providing the required hydrostatic support to the tubules. The custom bioreactor successfully provided an ex vivo platform that preserves naïve mouse kidney morphology and filtration for up to 7 days, although organ stress was observed. The positive results from this study indicate that the apparatus may be used for other bioengineering applications, including inducing disease states and interrogating tissue regeneration schemes as well as acellular kidney recellularization to potentially produce de novo kidneys.

## Figures and Tables

**Table 1:** Urine analysis. Urine was collected from the ureter cannula at day 7 from kidneys analyzed and compared to fresh high glucose DMEM medium and the medium in the reservoir at the time of the urine collection (20 h). The average of the reading and the range of the readings is presented. The live kidneys consumed protein and glucose during normal kidney metabolism as observed by the decrease in total protein, albumin and glucose values of the fresh medium compared to the medium in the reservoir at t = 20 h. In the glomeruli glucose and protein will pass from the perfusion medium (‘blood’) to the ultrafiltrate (‘urine’) but then be reabsorbed by the proximal tubule cells. In a healthy kidney almost all the protein and glucose is reabsorbed back into the blood. This is seen here with the levels of protein and glucose in the collected urine (Experimental) being much lower than the perfusion medium (Control 2).

Component Being Measured	Control#1 Fresh 10% FBS/DMEM/ High Glucose *n* = 1	Control#2 20 h. Perfusion Medium Collected from a Day 7 Kidney; 10% FBS/DMEM/High Glucose *n* = 1	Experimental Urine Collected from a Day 7 Kidney *n* = 3
Total Protein (mg/dL)	386.82	305.55	20.68 (range 9.68–28.74)
Albumin (mg/dL)	3.23	2.87	0.05 (range 0.01–0.11)
Glucose (mg/dL)	450	399.78	4.87 *n* = 2 (range 4.08–5.66)

## Data Availability

Not applicable.

## Supplementary Materials

The following supporting information can be downloaded at: https://www.mdpi.com/2073-4409/11/11/1822/s1, Supplementary Table S1: Complementary Error Function table. Supplementary Figure S1: PAS and TUNEL staining of an ex vivo perfused mouse kidney.
