# Correction: Won et al. Ex Vivo Perfusion Using a Mathematical Modeled, Controlled Gas Exchange Self-Contained Bioreactor Can Maintain a Mouse Kidney for Seven Days. *Cells* 2022, *11*, 1822

**DOI:** 10.3390/cells13080714

**Published:** 2024-04-19

**Authors:** Natalie Won, Jorge Castillo-Prado, Xinzhu Tan, John Ford, David Heath, Laura Ioana Mazilescu, Markus Selzner, Ian M. Rogers

**Affiliations:** 1 Lunenfeld-Tanenbaum Research Institute, Mount Sinai Hospital, Toronto, ON M5G 1X5, Canada; natalie_won@rogers.com (N.W.); jcastillo@lunenfeld.ca (J.C.-P.); xinzhutan@163.com (X.T.); 2 School of Biomedical Engineering, McMaster University, Hamilton, ON L8S 4K1, Canada; 3 Department of Physiology, University of Toronto, Toronto, ON M5S 1A8, Canada; 4 Department of Chemistry, University of Toronto, Toronto, ON M5S 3H6, Canada; ja.ford@utoronto.ca (J.F.); dave.heath@utoronto.ca (D.H.); 5 Department of General, Visceral and Transplantation Surgery, University Hospital Essen, University Duisburg-Essen, 45147 Essen, Germany; laura.mazilescu@uk-essen.de; 6 Soham & Shaila Ajmera Family Transplant Centre, University Health Network, Toronto, ON M5G 2C4, Canada; markus.selzner@uhn.ca; 7 Toronto General Hospital Research Institute, Toronto, ON M5G 2C4, Canada; 8 Department of Obstetrics and Gynaecology, University of Toronto, Toronto, ON M5G 1E2, Canada

In the original publication [1], there was an error in the legend of Figure 2B. The text in the manuscript’s Results section is correct, but the figure legend does not match. The correct legend appears below. The authors state that the scientific conclusions are unaffected. This correction was approved by the Academic Editor. The original publication has also been updated.

“(**B**) A mouse kidney was cannulated and perfused in the ex vivo perfusion system for 7 days. The oxygen levels were held to a narrow range of 5.7–6.3 ppm, indicating that the media was well-oxygenated throughout the kidney perfusion period.”
