# Supplementary material for: Ex Vivo Perfusion Using a Mathematical Modeled, Controlled Gas Exchange Self-Contained Bioreactor Can Maintain a Mouse Kidney for Seven Days
Source: Cells. 2022 Jun 2;11(11):1822. doi: 10.3390/cells11111822 (PMC9180119; doi:10.3390/cells11111822)
Supplement: Supplementary file 1 [file cells-11-01822-s001.zip › cells-1666313-supplementary.pdf]

Supplementary Table S1: Complementary Error Function table.

The complementary error function

η	erfc (η)	η	erfc (η)	η	erfc (η)	η	erfc (η)	η	erfc (η)	η	erfc (η)
0.00	1.00000	0.38	0.5910	0.76	0.2825	1.14	0.1069	1.52	0.03159	1.90	0.00721
0.02	0.9774	0.40	0.5716	0.78	0.2700	1.16	0.10090	1.54	0.02941	1.92	0.00662
0.04	0.9549	0.42	0.5525	0.80	0.2579	1.18	0.09516	1.56	0.02737	1.94	0.00608
0.06	0.9324	0.44	0.5338	0.82	0.2462	1.20	0.08969	1.58	0.02545	1.96	0.00557
0.08	0.9099	0.46	0.5153	0.84	0.2349	1.22	0.08447	1.60	0.02365	1.98	0.00511
0.10	0.8875	0.48	0.4973	0.86	0.2239	1.24	0.07950	1.62	0.02196	2.00	0.00468
0.12	0.8652	0.50	0.4795	0.88	0.2133	1.26	0.07476	1.64	0.02038	2.10	0.00298
0.14	0.8431	0.52	0.4621	0.90	0.2031	1.28	0.07027	1.66	0.01890	2.20	0.00186
0.16	0.8210	0.54	0.4451	0.92	0.1932	1.30	0.06599	1.68	0.01751	2.30	0.00114
0.18	0.7991	0.56	0.4284	0.94	0.1837	1.32	0.06194	1.70	0.01612	2.40	0.00069
0.20	0.7773	0.58	0.4121	0.96	0.1746	1.34	0.05809	1.72	0.01500	2.50	0.00041
0.22	0.7557	0.60	0.3961	0.98	0.1658	1.36	0.05444	1.74	0.01387	2.60	0.00024
0.24	0.7343	0.62	0.3806	1.00	0.1573	1.38	0.05098	1.76	0.01281	2.70	0.00013
0.26	0.7131	0.64	0.3654	1.02	0.1492	1.40	0.04772	1.78	0.01183	2.80	0.00008
0.28	0.6921	0.66	0.3506	1.04	0.1413	1.42	0.04462	1.80	0.01091	2.90	0.00004
0.30	0.6714	0.68	0.3362	1.06	0.1339	1.44	0.04170	1.82	0.01006	3.00	0.00002
0.32	0.6509	0.70	0.3222	1.08	0.1267	1.46	0.03895	1.84	0.00926	3.20	0.00001
0.34	0.6306	0.72	0.3086	1.10	0.1198	1.48	0.03635	1.86	0.00853	3.40	0.00000
0.36	0.6107	0.74	0.2953	1.12	0.1132	1.50	0.03390	1.88	0.00784	3.60	0.00000

Supplementary Figure S1: PAS and TUNEL staining of an ex vivo perfused mouse kidney.

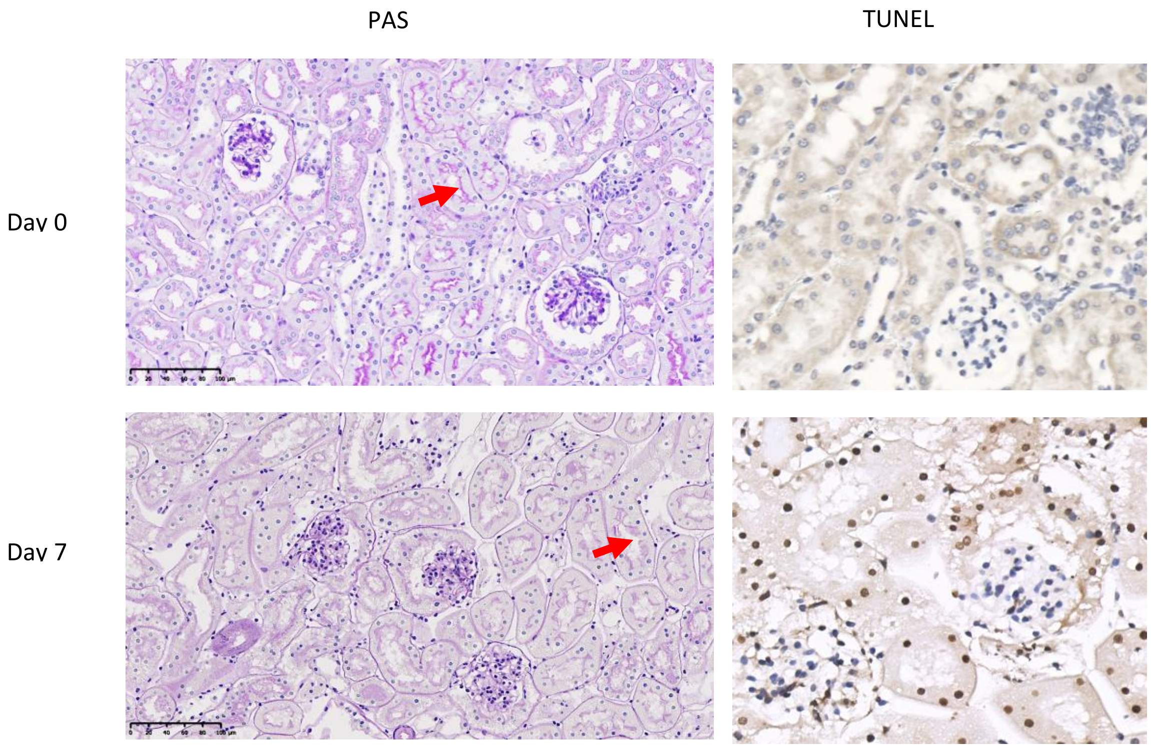

*Supplementary Figure S1:*Periodic-acid Schiff staining detects polysaccharides and highlights brush borders in renal tubular epithelial cells and basement membranes of glomerular capillary loops and tubular epithelium. Both day 0 and day 7 perfused kidneys show similar staining with PAS. Red arrow highlights brush border. TUNEL staining reveals that although the day 7 ex vivo perfused kidney shows normal PAS staining, Nephrin, LTL, KSP, AQP2 and E-cadherin staining, the tissue is under stress and apoptosis is initiated at day 7. The cells of the tubules seem to be more susceptible as they are stained positive (brown), while many cells of the glomeruli are heathy (blue).
